# ZTA Pipes with a Gradient Structure-Effect of the Rheological the Behavior of Ceramic Suspensions on the Gradient Structure and Characterized of the Obtained Products

**DOI:** 10.3390/ma14237348

**Published:** 2021-11-30

**Authors:** Radosław Żurowski, Justyna Zygmuntowicz, Paulina Piotrkiewicz, Marcin Wachowski, Michał M. Szczypiński

**Affiliations:** 1 Faculty of Chemistry, Warsaw University of Technology, 3 Noakowskiego Str., 00-664 Warsaw, Poland; 2 Faculty of Materials Science and Engineering, Warsaw University of Technology, 141 Wołoska Str., 02-507 Warsaw, Poland; paulina.piotrkiewicz@gmail.com; 3 Faculty of Mechanical Engineering, Military University of Technology, gen. S. Kaliskiego 2 Str., 00-908 Warsaw, Poland; marcin.wachowski@wat.edu.pl; 4 Department of Material Science, Faculty of Mechanical Engineering, Technical University of Liberec, Studenstká 2, 461 17 Liberec, Czech Republic; michal.szczypinski@tul.cz

**Keywords:** rheological properties, zirconia-alumina composites, centrifugal slip casting

## Abstract

This paper focuses on the verifying the possibility of producing Al_2_O_3_-ZrO_2_ composite pipes with a gradient structure using centrifugal slip casting method. The aim of the research is to define the correlation between the rheological properties of aqueous suspensions of ceramic powders with different solid loading and obtaining the ZrO_2_ phase gradient in the Al_2_O_3_ matrix. Such products, due to their unique properties, can be utilized in the transport of aggressive substances, even in extreme temperature or corrosive conditions. The suspensions and the sintered samples were characterized by: broad rheological analysis, scanning electron microscopy, X-ray diffraction, stereological analysis and Vickers hardness tests. The study reports on a series of samples produced of ceramic suspensions (70 vol.% Al_2_O_3_–30 vol.% ZrO_2_) differing in the total solid loading in the range of 30–55 vol.%. The results clearly indicate that obtaining the gradient structure of ceramic-ceramic composite pipes is closely related to the rheological properties of the suspensions from which the samples are cast. The phase gradient is obtainable from suspensions 30–40 vol.%, in which the possibility of moving ZrO_2_ particles relative to the Al_2_O_3_ is quite high—these suspensions are characterized by low viscosity and the dominance of viscous over elastic properties (G′ > G″).

## 1. Introduction

The high expectations set by society in almost every industry have led to tremendous progress in science [1]. As a result of the close-knit cooperation of researchers from all over the world and the resulting synergy, new fields of science have been created. One such example is, for instance, the field of composite materials. A skillful combination of at least two components can result in a product-composite with completely new or incomparably better functional properties in relation to the properties of the materials that were initially used to form it. In the 21st century, composite materials are present in almost every aspect of our lives and in nearly every area of industry. Ceramic-ceramic composites are particularly interesting. The combination of Al_2_O_3_ and ZrO_2_ enables the establishment of material Zirconia Toughened Alumina (ZTA) [2,3]. This is a composite material where alumina is toughened with zirconia. The principal advantage of Zirconia Toughened Alumina is the additional strength and toughness over alumina at a lower cost than zirconia. Therefore, ZTA materials are highly commercialized and marketed in hundreds of product types of different uses. The use of this type of material contributes an approving price-to-price ratio and unique mechanical properties. Currently, Zirconia Toughened Alumina is used as a common material for elements of devices and machines operating under friction and/or load conditions. 

Even though advanced ceramics have been widely used for many years, they are still of interest to researchers from all over the world. Great emphasis is placed primarily on the search for new or improvement of existing methods for forming ceramic products. The main objective is, of course, the widely understood improvement of the ecological aspects of the molding process, such as reduction or elimination of toxic substances, the use of additives which decompose to simple and harmless compounds during the sintering process, the use of water as a solvent or minimization of raw material losses [4,5,6,7,8]. It is also very important to make sure that ceramic materials receive in the forming process a shape that is as close as possible to that of the final product, and that parts with different, even complicated geometries, can be formed. 

One of the key processes in the manufacturing of ceramic materials is the formation stage. The selection of an appropriate method and the manner in which the process is carried out determines to a large extent the properties of the final product. Many methods of forming ceramic materials are known, which is excellently outlined in the work by Evans [9]. However, regardless which method is chosen as the most appropriate for a particular case, the rheological properties of the starting material, i.e., the powder, granulate, suspension or ceramic paste, are of the essence. 

One of the most common methods used in industry to form ceramic products is pressing (die pressing). As reported by Zipse [10], the main reason for its popularity is its relatively low cost. In addition, pressing does not generate large material losses and it allows for high density raw shapes to be obtained. However, in order for the formed shapes to exhibit the desired mechanical parameters, it is necessary to prepare the pressed material properly by furnishing it with appropriate rheological properties. One of the most common procedures used for this purpose is transformation of ceramic powder into granulate using the spray-drying process. The parameters of the granules thus obtained depend significantly on the rheological properties of the suspension and, above all, on its viscosity, whose value should be low enough for it to be atomized by thin nozzles [11,12]. 

Ceramic parts with slightly more complex geometries can also be obtained using the powder injection molding process. Ani et al. used this method to fabricate ZTA (zirconia toughened alumina) materials. They incorporated submicrometric alumina and zirconia powders with high-density polyethylene, paraffin wax and stearic acid as the molding charge. One of the key stages of the study was analysis of the rheological properties and homogeneity of the subsequently formed charge. This made it possible to select an appropriate composition including determination of the optimum ceramic powder content in the system [13]. 

Further proof of the usefulness of rheological measurements in forming ceramic materials with complex shapes is provided by the so-called novel dough forming process proposed by Seesal and Dhar [14]. This method, inspired by food technology of forming ceramic composites, is based on kneading a mixture of powders using a vinyl polymer until a mass with the desired visco-plastic properties is obtained. The resulting “ceramic cake” should exhibit non-Newtonian shear-thinning properties and very high viscosity, especially at low shear rates. In the cited publication, the actual viscosity of the prepared “cakes” ranged from 131.5 to 154.5 kPa·s. These “cakes” were suitable for extrusion above their yield strength of about 49–50 kPa, at a viscosity range of 45–55 kPa·s and a shear rate of about 1.25 s^−1^. The high plasticity index of the “cakes” ensured the replication of complex shapes with good dimensional retention. 

Preparation of a ceramic slurry that is suitable in terms of rheological properties, stability and homogeneity is also a key step in forming ceramic materials by tape casting or gelcasting methods [4,15,16]. In order to avoid problems while casting tape from a ceramic mass or pouring a slip into a mold, as well as to ensure good mold impression and good mechanical parameters of raw shapes, it is necessary to ensure a low viscosity and high solid phase content of the ceramic slurry. According to literature reports, in order to ensure good deaeration and slip settling, the suspension should exhibit shear thinning properties and its viscosity in the shear range from 10 to 100 s^−1^ should be no more than 1 Pa·s [17,18,19]. In addition to using rheological measurements to prepare suspensions with the desired viscosity, they can also be used to explain certain phenomena occurring in the gelling phase of the gelcasting method. Pietrzak et al. showed that the use of 2-carboxyethyl acrylate monomer in comparison with 2-hydroxyethyl acrylate led to a very significant reduction in oxygen inhibition during gelation, which in turn resulted in a lack of surface defects on the molded shapes. The reason behind the reduction of oxygen inhibition was found to be the very high viscosity value (above 1 kPa·s) of the aqueous Al_2_O_3_ suspension containing 2-carboxyethyl acrylate monomer as measured at low shear rates (below 0.5 s^−1^). Significantly higher viscosity in comparison with the suspension containing 2-hydroxyethyl acrylate limited oxygen penetration through the top layer, which ensured that polymerization occurred in nearly the entire sample volume [20]. On the other hand, Kedzierska-Sar et al. used rheological measurements to explain the mechanism of polymerization during gelcasting of a ceramic-metal system. They showed that in the obtained Al_2_O_3_-W materials, tungsten particles catalyzed the polymerization process, resulting in over 4 times lower gel activation energy at the surface of the metallic particles compared to the system without a metallic phase. In the slurry containing both alumina and tungsten, polymerization was initiated close to the surface of the metal particles, resulting in a heterogeneous polymer structure and poorer mechanical properties of the raw shapes [21].

The determination of rheological properties is also essential in the preparation of ceramic suspensions or pastes used in additive manufacturing techniques and 3D printing methods of ceramic materials. Halloran et al. demonstrated that the success of a stereolithography molding process depends on: rheological properties of the ceramic suspension, including its long-term stability and low viscosity, the value of which should not exceed several Pa·s [22,23]. On the other hand, the direct ink writing method (also known as robocasting) used for shaping materials utilizes ceramic pastes characterized by very high values of viscosity and yield stress as well as suitable viscoelastic properties [24,25,26]. Ceramic pastes are extruded through narrow nozzles layer by layer to produce structures with complex geometries. The preparation of pastes with the desired rheological properties ensures a successful printing process, which involves making sure that each successive layer does not melt and maintains the desired, previously designed shape [27]. According to literature reports, pastes exhibiting good printability should have a yield strength of not less than 10^2^ Pa and their G′ storage modulus should be at least 10^4^ Pa [28,29,30,31]. 

This publication is devoted to the fabrication of tube-shaped ceramic-ceramic composite materials with high application potential. Ceramic materials (ZTA, zirconia toughened alumina) were molded using the centrifugal slip casting (CSC) method, so that the shape of the raw product was almost identical to the sintered product. Given the fact that the CSC method produces elements with a rotationally symmetric cross-section, the results obtained and presented by the authors can be useful in the development of materials used as pipes for transporting aggressive and toxic media, even at elevated temperatures or pressures. We have already reported on the numerous advantages of using the CSC method and that it is suitable for the fabrication of composite ZTA pipes with good mechanical properties and with uniform distribution of zirconium oxide in the alumina matrix. Moreover, we have shown that this solution is environmentally justified [32,33,34]. The CSC method does not utilize large amounts of organic additives. It is beneficial from an ecological point of view, but not only from this viewpoint. The lack of a large number of organic substances significantly reduces the likelihood of numerous defects appearing during the sintering process. Moreover, the CSC method does not require the use of toxic substances, e.g., monomers necessary for the gel casting method. An additional advantage of the CSC method is also the sample formation time. It is much shorter compared to, for example, classic slip casting. 

In this manuscript we aim to focus our attention on how the rheological properties of ceramic CSC molded suspensions with different solid phase content influence the final properties of the fabricated tubes. Particular attention was paid to analyzing the correlation between the rheological properties of the suspensions and the possible gradient structure of the product. 

## 2. Materials and Methods

### 2.1. Test Materials

Alumina with the symbol TM_DAR (Taimei Chemicals Co., Ltd., Tokyo, Japan) and zirconium oxide with the symbol TZ-3YS-E (TOSOH Corporation, Tokyo, Japan) were used in the study. The ZrO_2_ powder was stabilized with 3 mol% yttrium oxide. The powders were chosen to follow up on previous research work on CSC-molded materials based on Al_2_O_3_ and ZrO_2_ [32,33,34]. Other substances used in this study included liquefying agents in the form of citric acid-CA (Sigma-Aldrich, anhydrous, ≥99.5%, St. Louis, MO, USA) and diammonium hydrogen citrate-DAC (Sigma-Aldrich, d.d.a., St. Louis, MO, USA), while a 10% solution of polyvinyl alcohol-PVA (PVA, Sigma-Aldrich, St. Louis, MO, USA) was used as a binder for sample fabrication. Deionized, MiliQ system purified water was used as a solvent. 

### 2.2. Preparation of Ceramic Casting Slips

A specific amount of solvent was prepared for the first step in the preparation of slips for rheological testing and for the subsequent stage of shape molding. The solvent was weighed and placed in sintered corundum containers. Afterwards, defined amounts of liquefiers (DAC and CA), binder and ceramic powders were added. After the addition of each substrate, the mixture was stirred with a glass dipstick to obtain a uniform consistency. The suspensions thus prepared were stirred in a Retsch PM 400 planetary ball mill (Retsch, Haan, Germany) at 300 rpm for 60 min. In the experiment, the slurries were mixed in an alumina jar. Five ceramic balls made of alumina oxide were used as the grinding media. The suspensions were then deaerated in a THINKY ARE 250 device (Thinky Co., Tokyo, Japan). The degassing process was carried out at 2200 rpm for 11 min. 

### 2.3. Measurement of Rheological Properties 

Viscosity measurements were performed using a Malver Instruments Kinexus Pro rotational rheometer (Malvern Panalytical, Malvern, Worcestershire, UK). The experiments were conducted in a plate-to-plate system with a 0.5 mm gap and executed first by increasing then by decreasing values of shear rate in the range from 0.01 s^−1^ to 100 s^−1^ and from 100 s^−1^ to 0.01 s^−1^. The viscoelastic properties of the ceramic suspensions were then determined based on the results obtained from the dynamic oscillatory measurements. In the first step, an amplitude scan was performed to determine the linear viscoelastic region (LVER) for each suspension—the sample was “swept” with an amplitude deformation range of 0.01% to 100% at a constant frequency of 1 Hz. In the next step, a variable frequency from 10 to 0.1 Hz was applied to the sample at a constant deformation value belonging to the LVER range. The values of G′-the storage (elastic) modulus and G″-the loss (viscosity) modulus were determined from the frequency test. The oscillatory measurements were carried out at 25 °C. 

### 2.4. Forming of Shapes by Centrifugal Slip Casting

Six series of specimens were produced, differing in the solid phase content of the casting slip used to form the shapes: Series I—30 vol.% solid phase with 30 vol.% ZrO_2_ and Series II—35 vol.% solid phase with 30 vol.% ZrO_2_, Series III—40 vol.% solid phase with 30 vol.% ZrO_2_ and Series IV—45 vol.% solid phase with 30 vol.% ZrO_2_, Series V—50 vol.% solid phase with 30 vol.% ZrO_2_ and Series VI—55 vol.% solid phase with 30 vol.% ZrO_2_. 

The composites were formed by means of the centrifugal slip casting method [32,33,34]. The method utilizes simple equipment design and an economical process, but requires very good knowledge of colloid chemistry and rheological properties of the casting slips. Therefore, the basic condition for the successful application of the centrifugal slip casting method is obtaining a stable suspension, as only then is it possible to obtain a gradient structure. Therefore, this paper is mainly devoted to the rheological study of the casting slips used to shape the samples by means of the CSC method. 

The first step consisted in the preparation of a homogeneous suspension, which was poured into a gypsum mold placed in a metal centrifuge housing and subjected to centrifugal casting in a specially prepared device. The CSC process was conducted at 3000 rpm for 110 min, each time at 25 °C. After molding, the sample, along with the gypsum mold, was removed from the device and dried for 48 h at 30 °C. In the next step, the shapes were removed from the gypsum mold and subjected to sintering. The sintering process was conducted in two different chamber furnaces. The first stage was carried out in a type RHF 14/15 Carbolite furnace, while the second stage in a type HTF 17/5 Carbolite furnace. A different heating program was used for each stage. The stages differed in heating rate and holding temperature. The sintering stages for the samples are summarized in Table 1. The first stage was conducted in order to remove moisture and ensure thermal decomposition of organic compounds, therefore it was executed at a slower heating rate to avoid defects on the surface of the shapes. In the second stage, the actual sintering of the shapes took place. 

### 2.5. Phase Composition Examination

A Rigaku Mini Flex II diffractogram (Rigaku, Tokyo, Japan) with CuKα radiation and a wavelength of λ = 1.5406 Å was used in the study. The investigations were performed using the following parameters: 2θ angular range—20°–100°, voltage—30 kV, current—15 mA, measurement step—0.01°, counting time—1 s. The diffractometric data was processed using MDI JADE 7 software (Materials Data Inc., Livermore, CA, USA). The ICDD PDF-4 + 2020 X-ray standard database was used to interpret the results. 

### 2.6. Apparent and Relative Density Examination

The relative density was evaluated via the hydrostatic method. For this purpose, the sintered pipes were cut into small parts with a height of about 0.5 cm. Then the samples were dried at 130 °C for 12 h. After cooling, they were weighed in air and then immersed in distilled water and boiled for 1 h. After that, the samples were weighed in distilled water and next, in air without removing water from the pores.

### 2.7. Microscopic Observations 

The raw powders were subjected to investigate size and shape in SEM analysis with HITACHI SU 8000 (Hitachi, Tokyo, Japan). The microstructure characterization of powder was carried out using BSE detector (Hitachi, Tokyo, Japan), voltage 5 kV, working distance 9.1, 9.2 and 9.3 mm with magnification from 1000 to 40,000 times. Moreover, fracture microstructure investigations were conducted to determine how the ZrO_2_ particles were distributed in the composites and to determine what effect changing the solid phase content of the casting slip had on the size and shape of the Al_2_O_3_ phase as a result of the sintering process. The experiments were performed using a JEOL JSM-6610 scanning electron microscope (JEOL Ltd., Tokyo, Japan). An accelerating voltage of 15 kV was applied. The observations of the polished cross-sections of the samples were carried out using an optical microscope Nikon ECLIPSE LV150N (Nikon Co., Tokyo, Japan).

### 2.8. Hardness Test 

The hardness of the produced composites was determined by the Vickers method using an HVS-30T hardness tester (Huatec Group Corporation, Beijing, China). The measurements were carried out on flat-parallel, metallographically prepared (grinding and polishing) cross-sections of sintered composite samples of each series. The impressions were made in a straight line, perpendicularly to the axis of specimen rotation, starting from the outer edge towards the core at a load of 98.1 N and an application load time of 10 s. The number of impressions made depended directly on the width of the specimen. In order to determine the hardness, the following Formula (1) was used:

(1) $$HV=\frac{0.1891F}{d^{2}}$$

where *F* corresponds to the load applied to the specimen in *N*, and *d* is the arithmetic mean of the diagonal lengths of the impression obtained after removal of the load in mm.

### 2.9. Stereological Analysis

A stereological analysis was carried out to determine the effect of the solid phase content of the casting slip on the alumina grain size after the sintering process. For this purpose, MicroMeter v.086b [35,36] software was used. Moreover, the analysis provided information on the shape of Al_2_O_3_ grains. 

## 3. Results

**Figure 1:**
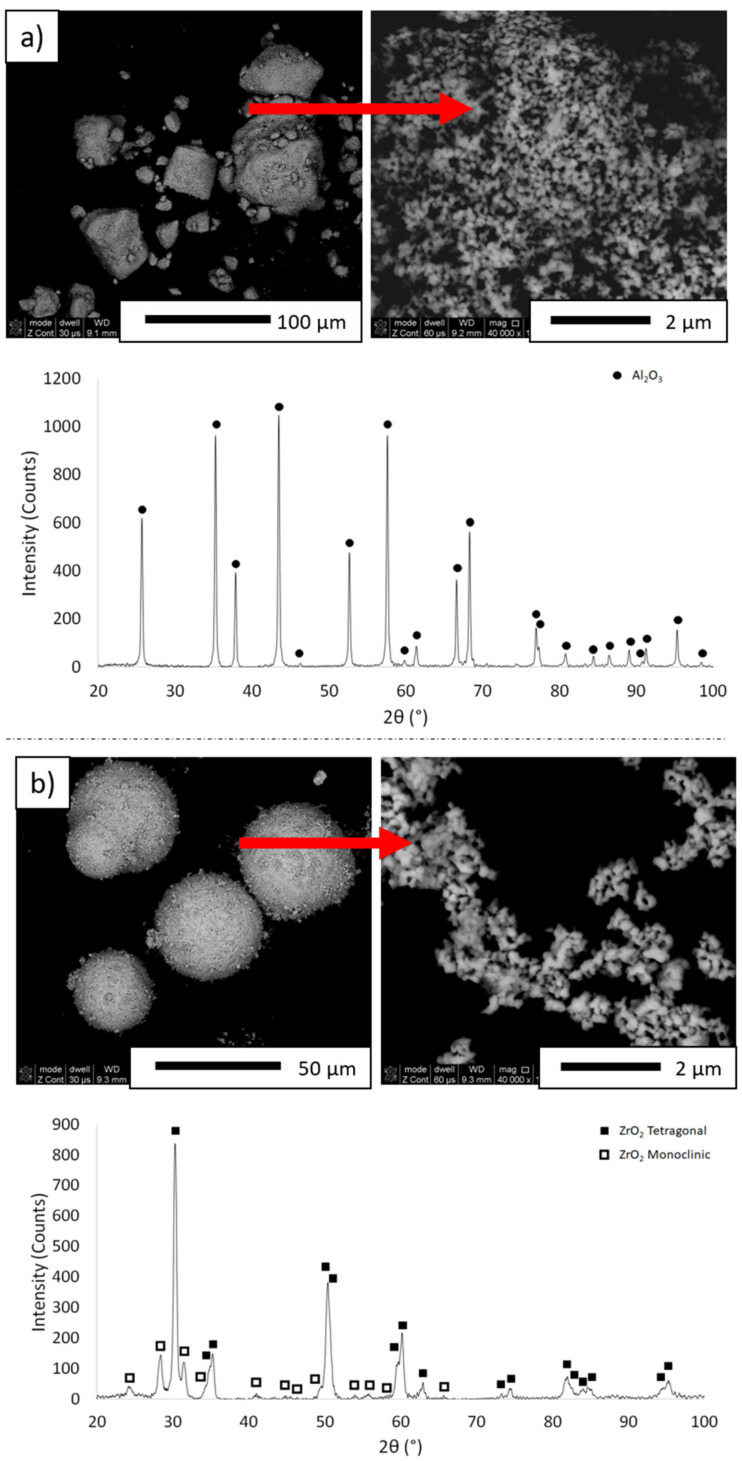
Morphology and XRD of the powders used in the experiment: (**a**) Al_2_O_3_, (**b**) ZrO_2_.

Aluminum oxide and zirconium oxide powder stabilized with 3 mol% of Y_2_O_3_ were used in the tests. According to the manufacturer’s data, α-Al_2_O_3_ powder has an average grain size of 0.12 ± 0.3 µm, while ZrO_2_ has an average grain size of 0.09 ± 0.25 µm. The microstructure and XRD of the powders used is shown in Figure 1. Based on the observations, the powders used tend to form agglomerates. This is justified as the manufacturer states that the powders are of submicronic and nanometric size and demonstrate a high tendency to agglomerate. The observations revealed that the ZrO_2_ powder forms >30 µm granules consisting of particles measuring 0.1 µm in size. From the diffractogram obtained for Al_2_O_3_ powder (Figure 1a), only peaks originating from α-Al_2_O_3_ [PDF #98-000-017] were observed. In the case of ZrO_2_ powder (Figure 1b), the presence of two phases was determined: tetragonal (PDF #00-050-108) and monoclinic (PDF #04-013-6617). The X-ray diffraction analysis accomplished revealed that the ZrO_2_ powder contained 63.7 wt.% of the tetragonal phase and 36.3 wt.% of the monoclinic phase.

**Figure 2:**
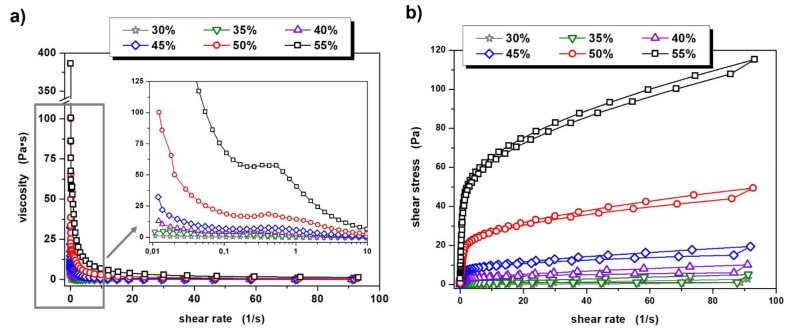
Viscosity curves (**a**) and flow curves (**b**) of prepared ceramic slurries.

The data presented in Figure 2a indicates that all of the prepared ceramic suspensions exhibit non-Newtonian flow characteristics in the analyzed shear rate range. The suspensions with a solid phase content of 30% and 35% by volume exhibit only shear-thinning properties. Other ceramic slips, i.e., those with powder concentrations between 40% and 55% by volume, also show a general shear thinning tendency, but in the low shear rate range (0.2–0.4 s^−1^) a slight increase in viscosity can also be observed. The origin for this phenomenon is likely to be the enhancement of hydrodynamic attractive forces causing the formation of small hydroclusters of powder particles in the suspension [37]. However, it should be noted that the recorded shear thickening effect in these cases is very small, with values ranging from 0.73 to a maximum of 1.82 Pa·s, depending on the suspension. These observations correspond well with our previous reports for similar aqueous Al_2_O_3_-ZrO_2_ suspensions of 50% by volume, in which the zirconium oxide phase was 5% and 10% [33]. It is worth noting, however, that the occurrence of shear thickening should not have negative effects at the later stage of sample formation. This is because the molding method used (centrifugal slip casting) induces much higher stress on a suspension than those at which shear thickening is observed. 

Further analysis of the results presented in Figure 2a. allows for the drawing of a rather obvious conclusion. The viscosity of the prepared suspensions increases with increasing solid phase content in the system and it is worth noting that these differences are significant. In the case of suspensions with a solid phase content of 30 vol.% and 35 vol.%, the initial viscosity is 1.2 and 4.5 Pa·s, respectively, while an increasing shear rate causes these values to drop to 0.03 and 0.06 Pa·s. Each subsequent 5 percent increase in the volume fraction of Al_2_O_3_ and ZrO_2_ powders in the suspension results in a three- to four-fold increase in the initial viscosity; thus, for ceramic slips with a solid phase “concentration” in the range 40–55%, these values amount to 13.2, 32.4, 100 and 386 Pa·s, respectively. Significant differences in viscosity between the prepared ceramic suspensions are of course also evident at increasing shear rates, as summarized in Table 2. 

The reason for the increase in viscosity of suspensions with higher solid phase content is the reduced average surface to surface separation distance between the particles (SDP) of the ceramic powders, and thus an enhancement of particle–particle interactions and friction between them. According to the study presented by Isobe et al., the SDP value in the case of aqueous Al_2_O_3_ suspensions decreases with increasing solid phase content in the system [38]. Based on Equation (2):

(2) $$DP=d\left[{\left(\frac{1}{3\pi \varphi }+\frac{5}{6}\right)}^{\frac{1}{2}}-1\right]$$

where: *d*–particle diameter; *φ*–volume fraction; and experimental data on the size of alumina particles in aqueous suspensions of different “concentrations”, researchers have shown that the SDP value increases up to about 10 nm, after which the particles merge into larger agglomerates keeping the SDP relatively constant. Needless to say, the initial Al_2_O_3_ size determines the threshold of ceramic powder content in the system over which changes in SDP values are observed. The use of smaller particle sizes causes their strong agglomeration already at lower powder contents in the system. Conversely, larger particles will start to agglomerate at their somewhat higher “concentration”. Interestingly, Isobe et al. used the same alumina powder in their study as the authors of this paper-Al_2_O_3_ TM-DAR. According to their research, a strongly enhanced agglomeration of this powder in an aqueous system is observed when its content exceeds 40%–45% by volume. Note that our systems differ slightly from those analyzed by Isobe et al.: in addition to Al_2_O_3_, ZrO_2_ was also used, and the volume ratio of these powders in each suspension is 70:30. Furthermore, apart from a slightly different dispersing agent, a small addition of PVA was also employed as a binder. The considerations presented here are relevant to the subsequent formation of tube-shaped ceramic shapes with a visible gradient of Al_2_O_3_ and ZrO_2_ phases. This is because the gradient at the molding stage can only be obtained when the viscosity of the suspension is fairly low, allowing the particles to move between each other relatively freely. Exceeding the powder concentration limit, after which the particles will agglomerate, will make this ease of movement much more difficult. 

The subsequent part of the conducted rheological measurements focused on the determination of thixotropic and viscoelastic properties of the prepared ceramic suspensions. Based on the obtained flow curves, it can be concluded that all of them are characterized by weak thixotropic properties, as evidenced by the occurrence of small hysteresis loops, which is presented in Figure 2b. 

**Figure 3:**
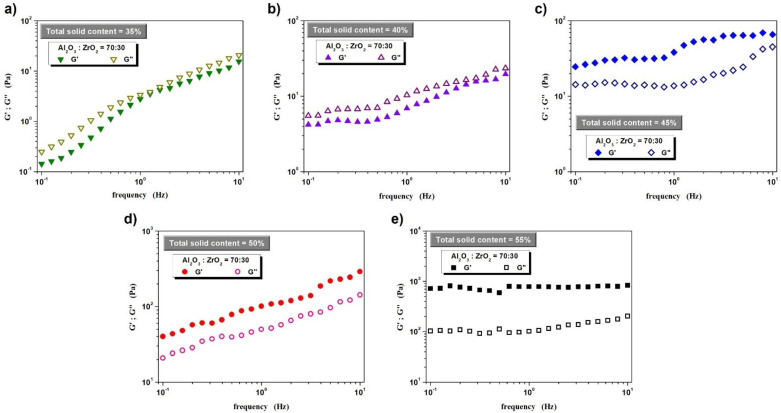
Correlation between the G′ storage modulus and G″ loss modulus as a function of frequency for ceramic suspensions with a solid phase content by volume of 35% (**a**); 40% (**b**); 45% (**c**); 50% (**d**) and 55% (**e**).

In turn, the results of dynamic oscillatory measurements, intended to determine the viscoelastic properties of the prepared slips, are presented in Figure 3. The analysis of the obtained data shows that in the case of suspensions with a solid phase content up to 40% by volume, viscous properties dominate (over elastic qualities): the value of the G′ storage modulus is lower than that of the G″ loss modulus in the entire frequency range investigated. This situation changes when the ceramic powder content in the system is at 45% by volume. Such a “concentration” of powder in the system suffices for an internal structure of the fluid to form, thus leading to the dominance of elastic properties. A further increase in the share of the solid phase does not change this situation and G′ dominates over G″, while a significant increase in values is observed, especially of the G′ storage modulus. 

In conclusion to this part of the study, it is important to stress that from a technological point of view, viscosity values of ceramic slips should also be measured at much higher shear rates, as the employed molding method (centrifugal slip casting) places very high stress on a suspension. As can be deduced from the data in Table 2, the viscosities measured at a shear rate of 100 s^−1^ for suspensions with a solid phase content between 30 vol.% and 55 vol.%, fall within the range of 0.03–1.24 Pa·s. It is worth pointing out, however, that each successive increase in the solid phase content by 5 vol.% in the range from 30 vol.% to 45 vol.% causes an almost twofold increase in the viscosity measured at $\dot{\gamma }$ = 100 s^−1^. In turn, when the solid phase content increases from 45 vol.% to 50 vol.% and from 50 vol.% to 55 vol.%, viscosity increases 2.5 times. This may suggest that the attainment of the Al_2_O_3_ and ZrO_2_ phase gradient at the molding stage of the tube-shaped ceramic shape can be achieved at 45% (and lower) solid phase content by volume. The viscosity of these suspensions at high stress is relatively low and the possibility of particles moving relative to each other appears to be rather undisturbed. It is worth remembering, however, that according to the results obtained from dynamic oscillatory measurements, a 45% volume content of the solid phase may cause difficulties in the movement of particles, whose accumulation in the system is already relatively high—so high that elastic properties dominate in the range of LVER deformations. The rheological tests carried out suggest that a gradient structure can be expected for the samples cast from suspensions with 30%, 35%, 40% and possibly 45% solid phase content by volume. 

**Figure 4:**
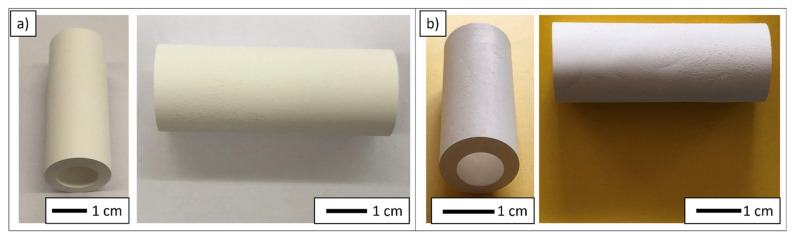
Example image of a CSC molded sample containing 35 vol.% solid phase: (**a**) before sintering, (**b**) after sintering.

The next stage focused on investigating the properties of the obtained molded shapes. The pieces fabricated using the centrifugal slip casting method have an axially symmetrical shape with a hole in the axis. The production of this type of material will make it possible to obtain so-called modular elements consisting of a number of tubular composite segments, which can be inserted into a single pipe, e.g., made of a metallic alloy. What is more, the obtained pipes will allow for transportation of molten metals, as such substances can only be transported through pipes made of ceramic materials, capable of operating at temperatures as high as 1000 °C. The presented solution makes it possible to manufacture tubular elements allowing for operation at such temperatures. Figure 4 shows an example of a CSC formed raw (Figure 4a) and sintered (Figure 4b) sample containing 35 vol.% solid phase. Analysis of the obtained photos shows that the shape lacks any apparent cracks or defects in the entire volume of the sample. The two-stage sintering process prevented the occurrence of deformations on its surface. It is worth noting that similar results were obtained for samples containing 40–55 vol.% solid phase. Unfortunately, for the sample with the lowest solid phase content (30 vol.%), it was not possible to obtain a product without cracks and surface delamination. Therefore, future investigations will be carried out to determine the appropriate rate of temperature rise and hold for molded pieces containing 30 vol.% solid phase.

**Figure 5:**
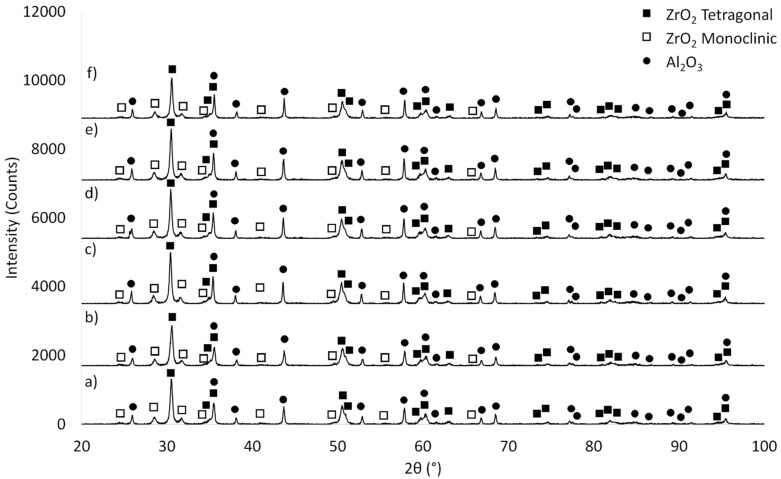
Phase composition analysis before sintering: (**a**) Series I—30 vol.% solid phase with 30 vol.% ZrO_2_, (**b**) Series II—35 vol.% solid phase with 30 vol.% ZrO_2_, (**c**) Series III—40 vol.% solid phase with 30 vol.% ZrO_2_, (**d**) Series IV—45 vol.% solid phase with 30 vol.% ZrO_2_, (**e**) Series V—50 vol.% solid phase with 30 vol.% ZrO_2_, (**f**) Series VI—55 vol.% solid phase with 30 vol.% ZrO_2_.

**Figure 6:**
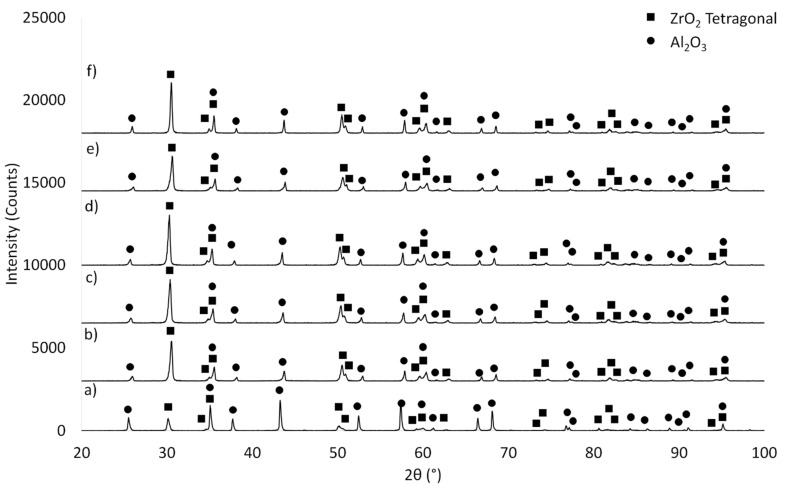
Analysis of the phase composition after the sintering process: (**a**) Series I—30 vol.% solid phase with 30 vol.% ZrO_2_, (**b**) Series II—35 vol.% solid phase with 30 vol.% ZrO_2_, (**c**) Series III—40 vol.% solid phase with 30 vol.% ZrO_2_, (**d**) Series IV—45 vol.% solid phase with 30 vol.% ZrO_2_, (**e**) Series V—50 vol.% solid phase with 30 vol.% ZrO_2_, (**f**) Series VI—55 vol.% solid phase with 30 vol.% ZrO_2_.

The next step focused on determining the phase composition of the composites before (Figure 5) and after the sintering process (Figure 6). The samples before and after sintering show significant differences in phase structure. In the samples before sintering (Figure 5), irrespective of the series studied, apart from Al_2_O_3_ (PDF #04-013-1687, #04-007-1400, #04-005-4505), tetragonal (PDF #04-005-4207, #00-050-1089, #00-068-0200) and monoclinic (PDF #01-072-0597, #04-013-6617, #04-013-1687, #04-004-4339, #04-013-4343) ZrO_2_ varieties can be found. On the other hand (Figure 6) reflexes from Al_2_O_3_ (PDF #98-000-0174, #00-005-0712, #04-008-3293, #04-006-2060, #00-010-0173) and the tetragonal ZrO_2_ (PDF #01-075-9645, #00-050-1089, #04-005-4207) phase occur in the sintered samples. 

The reason why the monoclinic ZrO_2_ variety is absent in the composites after sintering is explained by the permanence of the m-ZrO_2_ variety at low temperatures. Heating of samples containing m-ZrO_2_ to 1200 °C leads to its transition to the t-ZrO_2_ variety, which is what occurred in the conducted experiment [39,40]. The t-ZrO_2_ variety is stable up to a temperature of about 2370 °C [39,40]. Furthermore, the absence of reflexes from the m-ZrO_2_ phase in the sintered composites can also be attributed to the stabilization of the starting ZrO_2_ powder by an addition of 3 mol% Y_2_O_3_. The use of stabilized zirconium oxide allowed for the complete transition of the m-ZrO_2_ phase to t-ZrO_2_ during the sintering process at 1450 °C. 

Based on hydrostatic measurements, it was determined that all analyzed sintered samples were characterized by a very high density, which is the result expected with the used CSC forming method. Results obtained for Series II (35 vol.% Solid phase), Series III (40 vol.% Solid phase), Series IV (45 vol.% Solid phase), Series V (50 vol.% Solid phase) and Series VI (55 vol.% Solid phase) are 99.77 ± 0.13%, 99.71 ± 0.16%, 96.96 ± 0.49%, 97.57 ± 0.07% and 97.07 ± 0.17% respectively.

**Figure 7:**
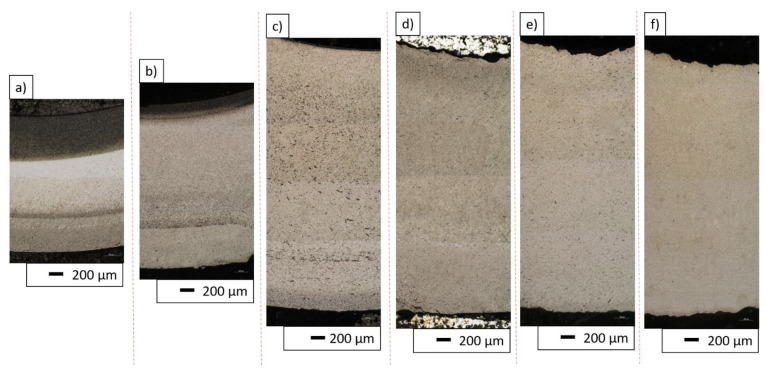
Optical images of the polished cross-sections of the samples (**a**) Series I—30 vol.% solid phase with 30 vol.% ZrO_2_, (**b**) Series II—35 vol.% solid phase with 30 vol.% ZrO_2_, (**c**) Series III—40 vol.% solid phase with 30 vol.% ZrO_2_, (**d**) Series IV—45 vol.% solid phase with 30 vol.% ZrO_2_, (**e**) Series V—50 vol.% solid phase with 30 vol.% ZrO_2_, (**f**) Series VI—55 vol.% solid phase with 30 vol.% ZrO_2_.

**Figure 8:**
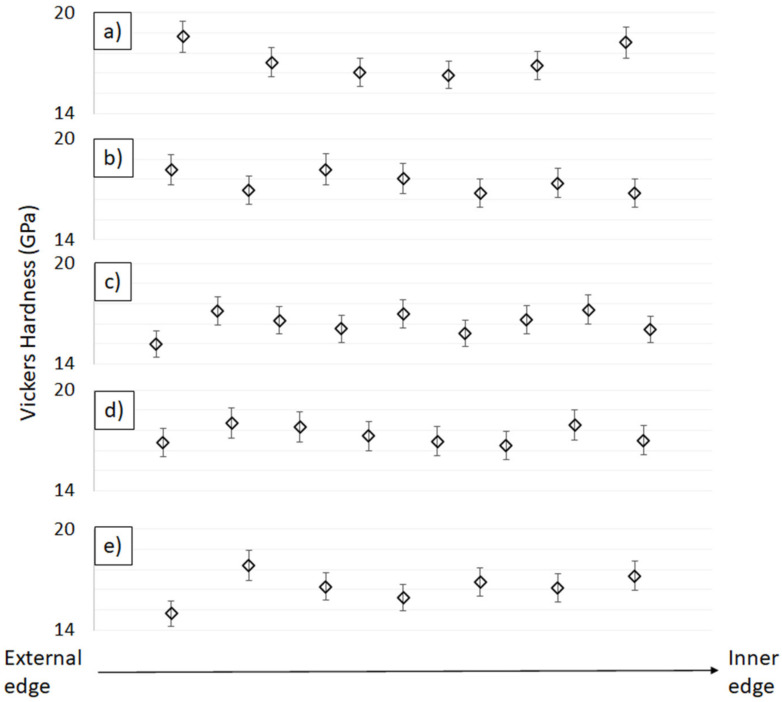
Hardness diagram for samples varying in solid phase content: (**a**) Series II—35 vol.% solid phase with 30 vol.% ZrO_2_, (**b**) Series III—40 vol.% solid phase with 30 vol.% ZrO_2_, (**c**) Series IV—45 vol.% solid phase with 30 vol.% ZrO_2_, (**d**) Series V—50 vol.% solid phase with 30 vol.% ZrO_2_, (**e**) Series VI—55 vol.% solid phase with 30 vol.% ZrO_2_. (For each measurement, the standard deviation is marked on the graph).

Using centrifugal force to cast components plays a key role in the formation of a second phase distribution gradient in the matrix structure. Optical images of the polished cross-sections of the samples shown in Figure 7 were analyzed in order to verify the presence of a gradient. Moreover, hardness tests were carried out linearly along the cross-sections of the samples of each of the produced series. The tests were carried out from the outer edge towards the core of each sample. The results are summarized in Figure 8.

All the sample series showed an identical volume content of ZrO_2_, while the average Vickers hardness results were in the range of 16–18 GPa. These values are lower than those obtained for pure Al_2_O_3_, whose hardness after sintering reaches values in the order of 20 GPa and higher [33]. This is directly related to the presence of a second ceramic component-ZrO_2_-with a much lower hardness, of the order from 12 to 16 GPa [33,41]. The use of a combination of these two components, despite the reduced hardness, has its justification in improving the material’s resistance to brittle fracture. In earlier studies published by the team, a deterioration in the hardness of Al_2_O_3_-ZrO_2_ composites compared to pure Al_2_O_3_ ceramics was observed [33]. However, the available scientific literature justifies the addition of ZrO_2_ as having a beneficial effect on the brittle fracture and flexural toughness of Al_2_O_3_ ceramics [41,42]. 

The analysis of the images from the optical microscope presented in Figure 7a–f clearly confirmed the presence of the gradient structure in the samples produced from the 30 vol.% and 35 vol.% series. An extended analysis of the results obtained for individual series revealed changes in the hardness of the material along its cross-section. These were particularly evident in samples containing 35 vol.% solid phase, which also confirmed the observed presence of a gradient in the structure of these composites. In the sample with 35 vol.% solid phase, the hardness changed symmetrically along the cross-section, and so the highest hardness was recorded at the ends, while the lowest was found in the central region of the cross-section. The reason for the high hardness value at the inner edge of the sample is the presence of a large accumulation of the Al_2_O_3_ phase in this region. The decrease in hardness from the inner to the central part of the cross-section of the sample can be explained by the increase in the proportion of the ZrO_2_ phase. The observed minimum in the central part and a renewed increase in hardness in the outer cross-section zone was initially quite an unexpected result. Even lower hardness was expected at the outer edge of the sample due to the accumulation of ZrO_2_ particles. However, it is worth noting, that during the stage of forming ceramic pipes using the CSC method, before the suspension in the gypsum mold is subjected to centrifugal forces, some time passes. This time is necessary to perform some process activities, such as transferring and putting the slurry in a plaster mold. The use of a plaster mold allows for removal of the water from the suspension via the capillary forces. It is the presence of the porosity in the plaster mold that causes the water to be absorbed from the ceramic, taking the shape of the mold and creating the cake in the first stage of the CSC process. The thickness of this “skin” is proportionate to the time for which it is permitted to form. Thus, in the created zone (“skin”), it is no longer possible for the particles to freely migrate during the formation by the CSC method. Therefore, at the outer edge of the pipe, a homogeneous ZTA composite can be expected, which would explain the increase again in hardness. For the sample with 40 vol.% solid phase content, a narrow area richer in Al_2_O_3_ phase was observed from the inner edge, followed by a slightly richer region in ZrO_2_. However, it is worth noting that the differences in phase imbalance are barely noticeable. The hardness results did not show any correlation, and the differences between the successive values are small. Therefore, it cannot be concluded on their basis that there is a clear gradient structure in the case of the 40 vol.% series. Nevertheless, numerous agglomerates of the Al_2_O_3_ phase were noticed in the cross-section of the sample. This means that the viscosity of 40 vol.% suspension may, on the one hand, be too high to create a significant phase gradient, and on the other hand, be low enough to allow the particles to move relatively freely in relation to each other. In the other samples with higher solid phase content, no significant disproportion in microscopic observations and hardness was observed along the cross-section and therefore no gradient structure was found. Only the first hardness values (at the outer edge of the sample) are very low (significantly lower than the others). The reason for this, however, may be that the impression is too close to the end of the sample. The sample with the lowest content of solid phase among those obtained (30 vol.% solid phase) was not subjected to hardness testing. The sample underwent significant delamination during production, which excluded it from further analysis. The delamination observed was most likely related to the insufficient solid phase content and the occurrence of strong segregation of the components in the sample area. Only a small fragment of the sample was embedded in resin to show the obtained microstructure gradient, which was shown in Figure 7a. As shown by the research conducted by S.K. Wang’s team, the solid phase content in the casting slip can strongly influence the gradient distribution in the structure of the cast composite. The solid phase content changes the viscosity of the casting slip contributing thus to reduced component segregation [43]. This phenomenon is the result of a combination of high variations in component density and high rotational speed during the centrifugal casting process. P. Rao’s team, in their work on Al_2_O_3_-ZrO_2_ composites, found that for a solid phase content in the composite below ca. 40 vol.%, segregation occurs resulting in an uneven distribution of phase components in the structure. They also discovered that increasing the solid phase content in the casting slip effectively eliminates this phenomenon [44]. 

Considering the multitude of factors influencing the properties of the composites obtained, which include: phase composition, the starting powders used and their processing methods, the sintering method, temperature, as well as the time of the sintering process, discrepancies in the results are inevitable. It should be noted, however, that the average hardness values obtained in this study for the produced Al_2_O_3_-ZrO_2_ composites with different solid phase contents are well in line with the trends presented in the available scientific literature on the subject. In the work by the team of C-Y. Huang, in which solid ZTA composites for ballistic applications were analyzed, composites with 30 vol.% ZrO_2_ produced via uniaxial pressing and sintered at 1550 °C were characterized by a hardness oscillating in the range of 12–14 GPa, which is lower than that of the materials produced in this study [41]. In the work by Ch. Meunier and his team, in which composites with a slightly higher content of ZrO_2_, equal to 40% by volume, were produced by a combination of uniaxial pressing and CIP (Cold Isostatic Pressing) and sintering with microwaves at 1500 °C, the measured hardness equaled ca. 17 GPa, which is a value similar to those obtained in this paper [45]. On the other hand, in a publication presented by D. Tang’s team, where ZTA composites with 30 vol.% ZrO_2_ content were also produced by uniaxial pressing and CIP (Cold Isostatic Pressing), but sintered freely at 1600 °C, the hardness obtained after the sintering process was ca. 20 GPa, a result that is slightly higher than that for the materials described [46]. 

**Figure 9:**
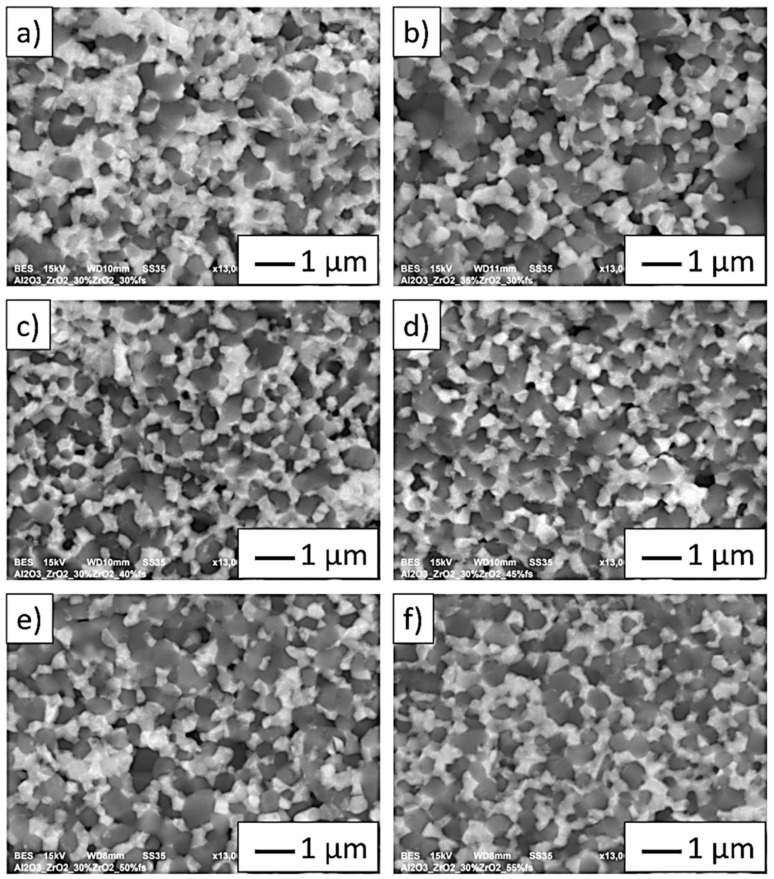
Sample fractographic observations of composites: (**a**) Series I—30 vol.% solid phase with 30 vol.% ZrO_2_, (**b**) Series II—35 vol.% solid phase with 30 vol.% ZrO_2_, (**c**) Series III—40 vol.% solid phase with 30 vol.% ZrO_2_, (**d**) Series IV—45 vol.% solid phase with 30 vol.% ZrO_2_, (**e**) Series V—50 vol.% solid phase with 30 vol.% ZrO_2_, (**f**) Series VI—55 vol.% solid phase with 30 vol.% ZrO_2_.

The SEM images shown in Figure 9a–f (in BSE mode) show characteristic areas of Al_2_O_3_-ZrO_2_ composites with different solid phase content in the casting slip used to form the samples (from 35 vol.% to 55 vol.%). In BSE mode, the zirconium oxide phase is shown as light grey areas, while alumina as dark grey areas. In all cases, only both phases of the composite were detected. SEM studies were performed for areas rich in the uniform distribution of ZrO_2_ in the Al_2_O_3_ matrix. It was observed that zirconium oxide particles do not form agglomerates in the alumina matrix. A fractographic examination of the specimens showed that weak bonds between the ceramic matrix particles-Al_2_O_3_ constituted the main sites of crack initiation. Similar results were observed for all the tested samples. The weak adhesion between the matrix particles is confirmed by the presence of voids in the microstructure, which form when matrix particles are torn out during cracking. The observation of cracks allows us to conclude that the Al_2_O_3_ matrix was characterized by grain decohesion when cracking occurred.

**Figure 10:**
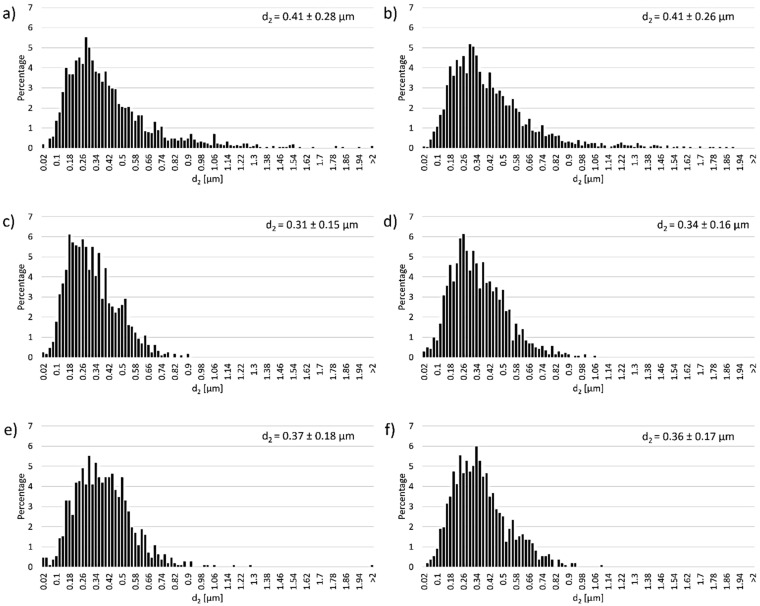
Histograms of Al_2_O_3_ grain size distribution depending on the solid phase content in the composites.

Figure 10 shows the histograms of Al_2_O_3_ grain size distribution depending on the solid phase content in the composites. The obtained histograms revealed that irrespective of the solid phase content in the casting slip, the samples exhibited monoclinic Al_2_O_3_ grain size distribution. The results show that samples containing 30 vol.% solid phase had an average Al_2_O_3_ grain size of 0.41 ± 0.28 µm. For these samples, Al_2_O_3_ grains were observed to be in the size range of 0.02 to 1.54 µm; however, single Al_2_O_3_ grains ranging in size from 1.54 µm to 2.05 µm were also present. Grains in the range of 0.28 µm to 0.4 µm had the highest frequency of occurrence. Similar grain size values for Al_2_O_3_ were recorded for samples containing 35 vol.% solid phase. For these samples (35 vol.%), the average Al_2_O_3_ grain size was 0.41 ± 0.26 µm, with the highest number of Al_2_O_3_ grains ranging from 0.22 µm to 0.42 µm in size. It was observed that for composites containing 40 vol.% and 45 vol.% solid phase, the obtained Al_2_O_3_ grain size values were slightly lower, but still within error limits. For samples with 40 vol.% solid phase content, the Al_2_O_3_ grain size ranged from 0.02 µm to 0.9 µm, with most grains measuring from 0.18 µm to 0.36 µm. The average Al_2_O_3_ grain size value for 40 vol.% was 0.31 ± 0.14 µm, while for 45 vol.% it was 0.34 ± 0.16 µm. The size range of Al_2_O_3_ grains for these samples (45 vol.%) was 0.02 µm to 1.06 µm, with most grains measuring from 0.18 µm to 0.38 µm. Samples with 50 vol.% and 55 vol.% solid phase content also showed slightly lower Al_2_O_3_ size values compared to samples with 30 vol.% and 35 vol.% solid phase, but still remained within error. For the 50 vol.% samples, an average Al_2_O_3_ grain size of 0.37 ± 0.18 µm was recorded. They ranged from 0.02 µm to 1.3 µm in size. The presence of single grains over 2 µm was also noted. For these samples (50 vol.%), most Al_2_O_3_ grains measured from 0.26 µm to 0.5 µm. However, for the 55 vol.% solid phase samples, the average Al_2_O_3_ size was 0.35 ± 0.17 µm. The grains measured from 0.02 µm to 1.1 µm in size, while the highest occurrence was recorded for the range from 0.24 µm to 0.4 µm.

Analysis of the histograms showed no significant effect of changing the solid phase content of the casting slip on the grain growth of Al_2_O_3_ during the sintering process of CSC-formed samples. 

In the next step, the Al_2_O_3_ grain parameter values were determined for shapes containing between 30 vol.% and 55 vol.% solid phase. Table 3 presents the results of the calculations, which showed that changes of the solid phase content in the casting slip do not affect the shape parameters of Al_2_O_3_ grains. All the determined parameters have similar values regardless of the tested sample. 

## 4. Conclusions 

In summary, the most important conclusions that can be drawn on the basis of the results presented in this paper include:

- Volume fraction of the solid phase (mixture of Al_2_O_3_ and ZrO_2_ ceramic powders) has a significant impact on the rheological properties of the aqueous-ceramic suspensions. The analysis of the viscosity curves showed that all of the prepared suspensions showed a non-Newtonian behavior with a general tendency to shear thinning. An increase in the solid loading significantly increases the viscosity of the suspensions: the greatest differences in the viscosity increasing were noted when changing the Al_2_O_3_/ZrO_2_ content from 45 vol.%. to 50 vol.% and 55 vol.%. Importantly, the oscillation tests indicated that the suspensions with a solid loading in the range of 30 vol.% to 40 vol.% are characterized by the dominance of viscous over elastic properties (G′ < G″). When the powder content is ≥45 vol.% the elastic properties dominate (G′ > G″), which proves the creation of a fairly strong 3D structure based on ceramic powder particle-ceramic powder particle and ceramic powder particle–water molecule interactions.
- Pipe-shaped green bodies were successfully produced from all prepared ceramic suspensions using the centrifugal slip casting method. Unfortunately, the sample formed from the suspension of 30 vol.% solid loading had very thin walls and cracked during the sintering process due to excessive stresses. The remaining green bodies (those obtained from suspensions with a solids loading of 35–55 vol.%) were successfully sintered. These samples are characterized by the absence of visible cracks and defects in their entire volume. The sintered samples were characterized by a very high density, from 97 to almost 100%.
- The microscopic observations and the hardness tests carried out linearly along the cross-sections of the sintered samples proved a gradient structure (ZrO_2_ phase gradient in the Al_2_O_3_ matrix) in the case of pipes formed by the CSC method from suspensions with a solid loading ≤ 35 vol.% and uneven distribution of both phases in case of 40 vol.%. With a higher solid phase content (≥45 vol.%), no gradient was observed and the ZrO_2_ grains were evenly distributed in the Al_2_O_3_ matrix. These results correlate well with the determined rheological properties of the suspensions. The limit value of the solid loading in the suspension is 45 vol.%, from which elastic properties dominate over viscous ones. In the case of suspensions with a solids loading ≥45%, strong interactions between their components are present. Moreover, these suspensions are characterized by a much higher viscosity compared to those in which the content of ceramic powders is 30–40 vol.% which is caused by the significantly reduced distances between the ceramic particles. All this means that the possibility of moving ZrO_2_ particles relative to the Al_2_O_3_ matrix is very limited and prevents the generation of a phase gradient even with the use of very high centrifugal force in the CSC method.
- The determined Vickers hardness of the produced ZTA pipe is in the range of 16–18 GPa, which is a satisfactory result and correlates well with other literature data.
- The performed stereological analysis did not show a significant influence of the change of the solid loading in the suspensions on the growth of Al_2_O_3_ grains in the sintering process of samples formed by CSC method.

## Figures and Tables

**Table 1:** Sintering stages.

First stage	heating	25 °C → 600 °C	2 °C/min
		600 °C → 1000 °C	5 °C/min
	holding	1000 °C	30 min
	cooling	1000 °C → 25 °C	5 °C/min
Second stage	heating	25 °C → 1450 °C	5 °C/min
	holding	1450 °C	120 min
	cooling	1450 °C → 25 °C	5 °C/min

**Table 2:** Dynamic viscosity values of the prepared ceramic suspensions for shear rates of 0.1, 1, 10 and 100 s^−1^.

Solid Phase Content	Viscosity at 0.1 s^−1^ Shear Rate (Pa∙s)	Viscosity at 1 s^−1^ Shear Rate (Pa∙s)	Viscosity at 10 s^−1^ Shear Rate (Pa∙s)	Viscosity at 100 s^−1^ Shear Rate (Pa∙s)
30%	0.30	0.08	0.04	0.03
35%	3.21	0.66	0.14	0.06
40%	4.22	2.51	0.44	0.11
45%	7.05	5.97	1.02	0.21
50%	18.6	14.5	2.80	0.53
55%	67.5	40.5	6.63	1.24

**Table 3:** Parameters describing shape factors of Al_2_O_3_ grains in the obtained samples.

Sample	Parameters Describing Shape Factors of Al_2_O_3_ Grains		
	Curvature of Grain Boundary R = p/(π·d_2_)	Elongation α = d_max_/d_2_	Convexity W = p/p_c_
Series I—30 vol.% solid phase with 30 vol.% ZrO_2_	1.27 ± 0.01	1.40 ± 0.01	1.08 ± 0.01
Series II—35 vol.% solid phase with 30 vol.% ZrO_2_,	1.29 ± 0.01	1.42 ± 0.02	1.09 ± 0.01
Series III—40 vol.% solid phase with 30 vol.% ZrO_2_,	1.25 ± 0.01	1.37 ± 0.01	1.08 ±0.01
Series IV—45 vol.% solid phase with 30 vol.% ZrO_2_	1.29 ± 0.01	1.42 ± 0.01	1.09 ± 0.01
Series V—50 vol.% solid phase with 30 vol.% ZrO_2_	1.29 ± 0.02	1.39 ±0.02	1.10 ± 0.01
Series VI—55 vol.% solid phase with 30 vol.% ZrO_2_	1.32 ± 0.05	1.40 ± 0.03	1.12 ± 0.03

Where: d_max_—maximum diameter of void projection [μm], d_2_—diameter of a circle of the same surface as the surface of the analyzed grain [μm], p—perimeter of void [μm], p_c_—Cauchy perimeter [μm] [35,36].

## Data Availability

Not applicable.
